# Critical illness among adults with cystic fibrosis in Texas, 2004–2013: Patterns of ICU utilization, characteristics, and outcomes

**DOI:** 10.1371/journal.pone.0186770

**Published:** 2017-10-24

**Authors:** Lavi Oud

**Affiliations:** Division of Pulmonary and Critical Care Medicine, Department of Internal Medicine, Texas Tech University Health Sciences Center at the Permian Basin, Odessa, Texas, United States; Azienda Ospedaliero Universitaria Careggi, ITALY

## Abstract

**Objective:**

Available reports on critically ill adults with cystic fibrosis (CF) suggest improving short-term outcomes. However, there is marked heterogeneity in reported findings, with studies mostly based on single-centered data, limiting generalizability. We sought to examine population-level patterns of demand for critical care resources, and the characteristics, resource utilization, and outcomes of ICU-managed adults with CF.

**Methods:**

We used the Texas Inpatient Public Use Data File to identify ICU admissions with CF aged ≥18 years in Texas between 2004–2013. We examined ICU utilization at population level (using CF Foundation annual reports) and, among ICU admissions, socio-demographic characteristics, burden of comorbidities, organ failure, life-support utilization and hospital disposition. Linear regression and multilevel logistic regression were used to examine temporal trends and predictors of short-term mortality (hospital death and discharge to hospice), respectively.

**Results:**

Of 9,579 hospitalizations of adults with CF, 1,249 (13%) were admitted to ICU. The incidence of ICU admission among adults with CF in Texas increased between 2004–2005 and 2012–2013 from 16.7 to 19.2 per 100 person-years (p = 0.0181), with ICU admissions aged ≥30 years accounting for 80.3% of the change. Among ICU admissions the following changes were noted between 2004–2005 and 2012–2013: any organ failure 30.2% vs. 56.3% (p = 0.0004), mechanical ventilation 11.5% vs. 19.2% (p = 0.0216), and hemodialysis 1.0% vs. 8.1% (p = 0.0007). Short-term mortality for the whole cohort and for those with mechanical ventilation was 11.4% and 41.8%, respectively, with corresponding home discharge among survivors 84% and 62.1%, respectively. Key predictors (adjusted odds ratios [aOR (95% CI)]) of short-term mortality included age ≥45 years (2.051 [1.231–3.415]), female gender (1.907 [1.237–2.941]), and mechanical ventilation (7.982 [5.001–12.739]).

**Conclusions:**

Adults with CF had high and rising population-level burden of critical illness. Although ICU admissions were increasingly older and sicker, the majority survived hospitalization, with most discharged home, supporting short-term benefits of critical care in the present cohort.

## Introduction

Cystic fibrosis (CF) is a heritable, life-shortening disease caused by dysfunction of the CF transmembrane conductance regulator, leading to progressive multisystem organ damage, with lung involvement being the predominant cause of morbidity and mortality. Remarkable strides were made over the past 2 decades in the care of CF patients, driven by advances in basic science, development of robust clinical trial network and multidisciplinary, evidence-based management in care centers, such as those accredited by the CF Foundation in the United States (US) [1, 2]. As a result, dramatic improvement was noted in long-term survival of CF patients, with over 50% being 18 year or older, reaching recently a predicted median survival of nearly 42 years [3], with projected median survival over 50 years, if the recently observed decrease in mortality continues at the same rate [4].

The increasing long-term survival into adulthood is expected to result in patients increasingly manifesting the cumulative impact of CF-, age-, and therapy-related morbidity [4]. Thus, the frequency of acute health crises, including those progressing to critical illness, can be expected to rise with the expanding adult CF population. The growing number of CF patients surviving into adulthood has in turn introduced challenges to maintain high quality of care as patients transition from pediatric to adult care systems, including the ICU [2, 4]. While earlier studies demonstrated the futility of intensive care and especially invasive mechanical ventilation in CF patients [5], more recent reports [6], showing improved outcomes among critically ill adults with CF, led to the proposition that critical care for CF “is no longer an oxymoron” [7]. Nevertheless, a debate continues about use of critical care [8] and invasive mechanical ventilation [9, 10] in adults with CF.

Data on the contemporary patterns of use of critical care services among adults with CF, and the characteristics and outcomes of ICU-managed patients can inform preventive and interventional efforts at the clinician and health system levels. However, data on the epidemiology and clinical features in these patients remains scant. There have not been, to our knowledge, population-based studies of critically ill adults with CF in the US. The investigations reported to date were based, for the most part, on singe-centered data with small cohort size, and the largest multicenter report included 136 ICU admissions [6]. The reported short-term mortality in these cohorts ranged widely from 18.3% [11] to 73% [12] for all critically ill patients, and from 0% [13] to 100% [14] of those undergoing invasive mechanical ventilation, though the majority of studies on the later subgroup reported mortality rates between 32.3% [15] and 58% [11]. Thus, the findings of the available studies may not be generalizable, and most reports on ICU-managed adults with CF described cohorts from as late as the early 2000s [6, 11, 12, 14–16]. In addition, given the sparsity of contemporary data, combined with lack of longitudinal studies, it is unknown whether general improvements in critical care and advances in CF-related management extend to outcomes of critically ill adults with CF.

The objectives of the present study were to conduct a population-level examination of adults with CF to evaluate the a) temporal patterns of utilization of critical care services b) characteristics, resource utilization and outcomes of those admitted to ICU and c) patient- and hospital- level factors associated with short-term mortality. We studied the CF population in Texas, a state with a large, demographically diverse population, with a high-quality administrative data that systematically reports on admission to ICU.

## Materials and methods

This was a retrospective, population-based cohort study. The study was determined to be exempt from formal review by the Texas Tech Health Sciences Center’s Institutional Review Board due to use of a publicly available, de-identified data set.

### Data sources and study population

We used the Texas Inpatient Public Use Data File (TIPUDF), an administrative data set maintained by the Texas Department of State Health Services [17].The use of TIPUDF has been previously described [18]. Briefly, the data set includes de-identified inpatient discharge data on the demographic, clinical, resource utilization, and outcome domains from state-licensed, non-federal hospitals, and captures 93% to 97% of all hospital discharges in the state. The state of Texas masks gender and zip code data of hospitalizations with a diagnosis of infection with the human immunodeficiency virus (HIV), ethanol or drug abuse; in addition, zip code data are masked in specific categories of hospitalizations with small number from a given zip code of residence or small number discharges from a specific hospital. In addition, the state provides only broad age group data for hospitalizations with a diagnosis of infection with the human immunodeficiency virus (HIV), or with alcohol or drug abuse, to protect patient privacy. Thus, age groups for the latter category of hospitalizations are reported as 18–44 years, 45–64 years, 65–74 years, and ≥75 years. TIPUDF provides discharge-level, rather than patient-level information, precluding accounting for repeated admissions in the data set. We thus report number of hospitalizations and ICU admissions as units of analysis, rather than number of patients.

We identified hospitalizations of state residents with CF, aged ≥18 years during the years 2004–2013, using International Classification of Diseases, Ninth Revision, Clinical Modification codes 277.0X. CF hospitalizations with transplantation of any organ were excluded. Hospitalizations with ICU admission served as the primary analytic cohort.

### ICU admissions

Hospitalizations with ICU admission were identified based on unit-specific revenue codes for an intensive care unit or a coronary care unit. Intermediate care units or step-down units were not included. Because administrative data sets do not include information of the temporal course of hospitalization, it could not be determined whether ICU admissions occurred at the start of hospitalization or later during hospital course. Similarly, the proximate indications for ICU admission were not available.

### Outcomes

Our primary outcome measure was short-term mortality of ICU-admitted hospitalizations, defined as the combination of hospital mortality and discharge to hospice. Secondary outcomes included temporal patterns of ICU utilization, burden of chronic illness, organ failure, use of life-support interventions, resource utilization, and hospital disposition.

### Study variables

We accounted for patient characteristics and hospital attributes. Patient-level covariates of ICU admissions included: a) demographics (age, gender, race/ethnicity [categorized as non-Hispanic black (black), non-Hispanic white (white), Hispanic, and Other], health insurance [categorized as Private, Medicare, Medicaid, Uninsured, and Other], rural residence [defined as ≥50% rural population at the zip code of residence [19]], and median income at the zip code of residence, based on US Census data [20] [reported as quartiles]) b) comorbid conditions, based primarily on the Deyo modification of the Charlson Comorbidity Index [21, 22]; in addition, we collected data on reported depression, anxiety, alcohol and drug abuse, and tobacco use [S1 Table], and malnutrition [23]); we reported broad age categories for the whole cohort in order to provide full representation of study population since, as noted above, the state of Texas suppresses more granular age data to protect the privacy of those with diagnoses of infection with HIV, or with ethanol or drug abuse; additional data on age group distribution were provided for those ICU admissions without the aforementioned 3 diagnosis categories c) medical or surgical hospitalization (based on the primary diagnosis-related grouping) d) organ failures [24] e) use of invasive mechanical ventilation (termed mechanical ventilation hereafter), hemodialysis, and blood transfusion [S1 Table] f) hospital disposition [categorized as routine home, home with home health, transfer to another hospital, transfer to a nursing facility, death, hospice, and a leave against medical advice] and g) year of hospitalization.

Hospital-level covariates included the total number of hospital beds, ICU capacity (fraction of total beds that are ICU), teaching status, rural designation, and whether the facility was accredited as part of the CF Foundation Care Center Network. The American Hospital Association’s Annual Survey data from 2004 through 2013 were used to ascertain hospitals’ total and ICU bed numbers. Teaching status was derived from TIPUDF facility designation. Rural designation of hospitals was obtained from the Texas State Office for Rural Health [25] and CF Foundation Care Center Network designation was obtained from Foundation’s website [26].

In addition, we collected data on hospital length of stay and total hospital charges of ICU admissions. Total hospital charges were adjusted for inflation using the consumer price index and reported in 2013 US dollars [27]. TIPUDF and the state of Texas do not provide tools for conversion hospital charges to costs.

### Data analysis

We summarized patient and hospital characteristics as numbers and percentages for categorical variables, while continuous variables were reported as mean (standard deviation [SD]) or median (interquartile [IQR]). Because prior reports on utilization of hospital resources generally provided data on the means of abnormally distributed variables, we reported both mean and median data to allow comparisons. We used the chi-square test for group comparison involving categorical variables and Cochran-Armitage test for trend, as appropriate. The t-test, Mann-Whitney test, and Kruskall-Wallis test were used for comparison of continuous variables, as appropriate. The Cochran-Armitage test for trend for used for categorical variables and the Jonckheere-Terpstra trend test was used with Kruskal-Wallis comparisons.

We examined ICU utilization as incidence of ICU admission among the adult CF population in Texas, the rate of ICU admission among all hospitalized adults with CF, and the rate of ICU admission across age strata of CF hospitalizations. We used the incidence of ICU admission as a proxy measure of the burden population-level critical illness. To estimate the number of adults with CF in Texas during each study year we first obtained the state-specific number of those included in the CF Foundation’s Patient Registry, using the Annual Reports by the CF Foundation [28]. Because it has been estimated that the Patient Registry includes 83% to 87% of the CF population in the United States [4, 19, 29, 30], we used the mid-estimate of 85% with the coefficient of 1.18 to derive an estimate of all adults with CF in the state for each year, similar to the approach described by Hamosh and colleagues [29]. The incidence of ICU utilization among the adult CF population in the state is reported as number of ICU admissions per 100 person-years.

We used 2-year time periods (2004–2005, 2006–2007, 2008–2009, 2010–2011, and 2012–2013) for group comparisons, trend analyses, and to examine for secular trends of short-term mortality in logistic regression analyses. Two-year time periods were used in order to increase group size and power for detection for statistically significant differences.

We used linear least squares regression of log-transformed data to examine the temporal trends of ICU utilization, comorbidity burden, development of any organ failure and number of failing organs, resource utilization, and hospital disposition. Trends were reported as average biennial percent change and corresponding 95% confidence intervals (95% CI). For trend analysis of hospital disposition we combined outcomes into the following categories: short-term mortality (as defined earlier), home (combined home and home with health services), and discharge to another facility (combined discharge to another hospital and to a nursing facility).

Because temporal changes in ICU utilization may be affected by changes in threshold of ICU admission, rather than changing needs, we examined the corresponding temporal trends of measures of severity of illness, using the number of organ failures. Because temporal changes in reported organ failure may reflect upcoding and reimbursement-related incentives [31], we further compared the rates of utilization of organ-specific life-support interventions among ICU admissions with a specific organ failure (i.e., use of mechanical ventilation among hospitalizations with reported respiratory failure and hemodialysis among those with acute renal failure) at the start and end of study period.

We used multivariate logistic regression modeling to examine predictors of short-term mortality of ICU admissions. Univariate logistic regressions were carried out, with covariates with p < 0.1 considered for multivariate analysis. Candidate covariates were then examined for multicollinearity. The multivariate logistic model included the following covariates: age, gender, race/ethnicity, congestive heart failure, malignancy, metastatic tumor, drug abuse, malnutrition, organ failure, blood transfusion, hemodialysis, mechanical ventilation, and teaching status of facilities. Year of hospitalization (as 2-year time periods) was used as adjustment covariate. Covariates were entered using backward stepwise selection. Model’s discrimination was assessed as area under receiver operating characteristic (AUROC) and its calibration by using the Hosmer-Lemeshow’s goodness-of-fit test. Because disparity in long-term survival among adult women with CF was recently reported to vary across age groups, with similar long-term survival among older patients [32], we checked for interaction between gender and age to assess whether similar patterns are observed for short-term mortality and included the interaction term in the final model. We further performed exploratory analyses to compare short-term gender-specific mortality rates among ICU admissions across age strata. We reported model findings as adjusted odds ratios (aOR) and their corresponding 95% CI.

Data management was performed using Excel and Access (Microsoft, Redmond, Washington) and statistical analyses were performed with MedCalc version 17.5.5 (MedCalc Software, Ostend, Belgium) and SAS version 9.3 (SAS Institute, Cary, North Carolina). A 2-sided *p* value < 0.05 was considered statistically significant.

## Results

### ICU utilization patterns

There were 9,579 hospitalizations of adults with CF during study period, of which 1,249 (13%) were admitted to ICU. The biennial number of hospitalizations of adults with CF and the corresponding number of ICU admissions are detailed in S2 Table.

**Fig 1:**
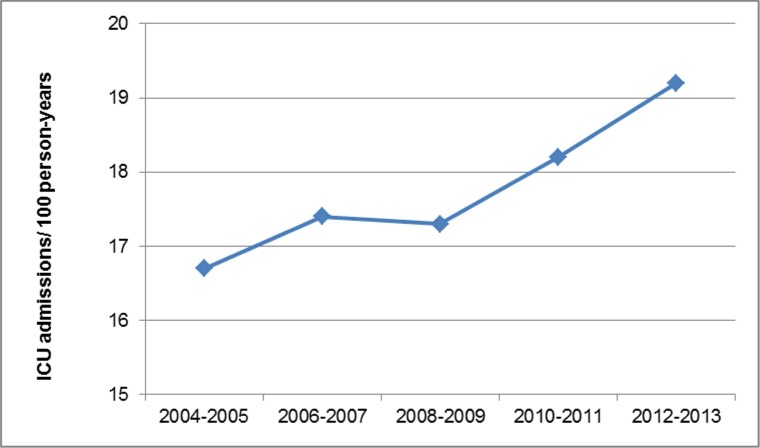
The biennial incidence of ICU admission among the adult population with cystic fibrosis in Texas, 2004–2013.

The temporal trends of the incidence of ICU admissions among the adult CF population in Texas are presented in Fig 1. The number of ICU admissions among adults with CF in the state between 2004–2005 and 2012–2013 rose from 16.7 to 19.2 per 100 person-years (+3.2%/2-years [95% CI 1.0% to 5.4%]). The rate of ICU admissions among hospitalized adults with CF did not change significantly over time (+3.6%/2-years [95% CI -2.1 to 9.3]). The rate of ICU admission among CF hospitalizations rose with age, being 11.8%, 22.9%, and 32.4% for age groups of 18–44 years, 45–64 years and ≥65 years, respectively (p < 0.0001 for trend). The number of ICU admissions increased from 192 in 2004–2005 to 334 in 2012–2013, with 80.3% of the change accounted for by ICU admissions aged ≥30 years.

### Characteristics, resource utilization, and outcomes of ICU admissions

The characteristics of ICU admissions are detailed in Table 1. Hospitalizations aged ≥45 years accounted for 17.1% of ICU admissions, while racial and ethnic minorities comprised 28%. The age distribution among ICU admissions without diagnoses of HIV infection, alcohol or drug abuse (n = 1,154) aged 18–29 years, 30–44 years, 45–64 years, and ≥65 years was 64.4%, 17.8%, 11.1%, and, 6.7%, respectively. Health insurance by Medicare or Medicaid was used by half of ICU admissions. Non-pulmonary comorbidities (among those included in the Deyo comorbidity index) were reported in 26.2% of ICU admissions, with diabetes (24.1%), liver (11.8%) and renal (9.0%) diseases being the most common. Between 2004–2005 and 2012–2013 there has been no change in the frequency of diabetes (22.4% vs. 20.7%; p = 0.6397), while occurrence of renal (3.1% vs. 14.4%; p < 0.0001) and liver (9.4% vs. 16.6%; p = 0.0293) disease rose substantially. Malnutrition was reported in 29.5% and depression and/or anxiety in 22.6%. Alcohol and drug abuse were uncommon and tobacco use was reported in 7.8%. HIV infection was reported in two ICU admissions. One or more organ failures occurred in 44.6% of ICU admissions (mean [SD] 0.7 [0.9]), with the respiratory (29.2%) and renal (18.9%) systems most commonly affected. The median (IQR) number of organ failures and Deyo comorbidity index scores increased across the 18–44 years, 45–64 years and ≥65 years age groups (0 [0–1], 0.5 [0–1], and 1 [0–1]; p = 0.0005 and p = 0.0027 for trend; 1 [0–1], 1 [1–3], and 2 [1–3]; p < 0.0001 and p < 0.0001 for trend, respectively). When ICU admissions without diagnoses of HIV infection, alcohol or drug abuse were examined, their median (IQR) number of organ failures and Deyo comorbidity index scores increased across the 18–29 years, 30–44 years, 45–64 years, and ≥65 years age groups (0 [0–1], 0 [0–1], 0.5 [0–1], and 1 [0–1]; p = 0.000 and p = 0.0007 for trend; 1 [0–1], 1 [0–1], 1 [1–3], and 2 [1–3]; p < 0.0001 and p < 0.0001 for trend, respectively). Mechanical ventilation and hemodialysis were used in 17.1% and 4.9% of all ICU admissions, respectively. When examined among ICU admissions with respiratory and renal system organ failures, mechanical ventilation and hemodialysis were used in 58.4% and 25.8%, respectively. The median (IQR) hospital length of stay and adjusted total hospital charges were markedly higher among ICU admissions who had mechanical ventilation, as compared to those who had not (14 [8–26] vs. 8 [4–14 days]; p < 0.0001 and $180,410 [90,290–375,345] vs. $66,359 [32,269–137,301; p < 0.0001, respectively). The corresponding mean (SD) lengths of stay and adjusted total hospital charges among ICU admissions who had mechanical ventilation, as compared to those who had not were 20.4 (23.4) vs. 11.0 (10.8) days and $265,961 (276,821) vs. $118,280 (154,714), respectively.

**Table 1:** The characteristics, hospital resource utilization, and outc0mes of ICU admissions.

	ICU Admissions
Variables	n = 1,249
**Age, years (%)**	
18–44	1,035 (82.9)
45–64	134 (10.7)
≥ 65	80 (6.4)
**Gender (n = 1,152 [%])**^**A**^	
Female	559 (48.5)
**Race/ethnicity (%)**	
White	899 (72.0)
Hispanic	199 (15.9)
Black	73 (5.8)
Other	78 (6.2)
**Median income (n = 1,114)**^**A**^	
Mean (US dollars)	44,925
Quartile 1 (lowest)	612 (54.9)
Quartile 2	423 (38.0)
Quartile 3	68 (6.1)
Quartile 4 (highest)	12 (1.0)
**Rural residence (1,114) (%)**^**A**^	146 (13.1)
**Health insurance (%)**	
Private	512 (41.0)
Medicare	314 (25.1)
Medicaid	319 (25.5)
Uninsured	63 (5.0)
Other	26 (2.1)
Missing	15 (1.2)
**Deyo comorbidity index (mean [SD])**	1.2 (1.6)
**Non-pulmonary comorbidities (%)**	
Any^B^	326 (26.2)
Diabetes^C^	301 (24.1)
Liver disease^C^	147 (11.8)
Renal disease^C^	113 (9.0)
Congestive heart failure^C^	100 (8.0)
Malignancy^C^	25 (2.0)
Malnutrition	369 (29.5)
Alcohol abuse	37 (3.0)
Drug abuse	75 (3.0)
Smoking	98 (7.8)
Anxiety	115 (9.2)
Depression	212 (17.0)
**Type of hospital admission (%)**	
Medical	944 (75.6)
Surgical	305 (24.4)
**Number of organ failures (%)**	
0	692 (55.4)
1	368 (29.5)
2	119 (9.5)
3+	70 (5.6)
**Type of organ failures (%)**	
Respiratory	365 (29.2)
Cardiovascular	69 (5.5)
Renal	236 (18.9)
Hepatic	29 (2.3)
Hematological	105 (8.4)
Metabolic	98 (7.8)
Neurological	47 (3.8)
**Mechanical ventilation (%)**	213 (17.1)
**Hemodialysis (%)**	61 (4.9)
**Blood transfusion (%)**	211 (16.9)
**Hospital length of stay (days)**	
Mean (SD)	12.6 (14.2)
Median (IQR)	9 (4–16)
**Total hospital charges**	
Mean (SD)	143,465 (189,620)
Median (IQR)	79,394 (37,745–174,073)
**Hospital disposition (%)**	
Routine home	774 (62.0)
Home with home health	160 (12.8)
Another hospital	127 (10.2)
Nursing facility	24 (1.9)
Leave against medical advice	21 (1.7)
Death	126 (10.1)
Hospice	16 (1.3)
Missing	1 (< 0.1)

A Gender was masked in 97 ICU admissions; Zip codes were masked in 135 of ICU admissions

B Comorbidities other than chronic lung disease within those included in the DEyo comorbidity index

C Part of the Deyo comorbidity index

The number of ICU admissions across examined hospital attributes is described in Table 2. Over 70% of ICU admissions were in hospitals with 400 or more beds and 50.5% occurred in teaching facilities. ICU admissions in rural hospitals and those that were part of accredited CF Foundation Network Care Center were uncommon.

**Table 2:** ICU admissions according to hospital characteristics.

	ICU Admissions
Hospital characteristic	n = 1,249
**Bed numbers (%)**	
< 200	119 (9.5)
200–399	248 (19.9)
400–599	440 (35.2)
≥600	442 (35.4)
**ICU capacity (%)**	
< 10	254 (20.3)
10–15	733 (58.7)
> 15	262 (21.0)
**Teaching facility (%)**	631 (50.5)
**CFF Care Center (%)**^A^	163 (6.2)
**Rural hospital (%)**	21 (1.7)

CFF: Cystic Fibrosis Foundation

A A facility that is part of the Cystic Fibrosis Foundation-accredited care centers

The short-term mortality for the whole cohort was 11.4% and after excluding those, 84% of the remainder were discharged home. Among the 213 ICU admissions requiring mechanical ventilation, short-term mortality was 41.8%. Among the remainder (n = 124), 62.1% were discharged home, 35.5% were discharged to another hospital, and 2.4% were discharged to a nursing facility. When analyzed across age groups, short-term mortality for age groups 18–44 years, 45–64 years, and ≥65 years was 10.4%, 13.4%, and 20.0%, respectively (p = 0.0078 for trend). The corresponding short-term mortality for age groups 18–29 years, 30–44 years, 45–64 years, and ≥65 years among ICU admissions without diagnoses of HIV infection, alcohol or drug abuse, was 9.7%, 15.6%, 14.1%, and 20.8%, respectively (p = 0.0012 for trend).

### Temporal trends among ICU admissions

The mean Deyo-Charlson index increased from 0.9 to 1.5 between 2004–2005 and 2012–2013 (+14.3%/2 years [95% CI 2.9% to 25.7%]). Occurrence of one or more organ failures rose from 30.2% to 56.3% between 2004–2005 and 2012–2013 (+16.3%/2 years [95% CI 13.3% to 19.3%]) and the mean number of failing organs increased from 0.4 to 0.9 over the same period (+20.4%/2 years [95% CI 13.1% to 27.7%]).

The frequency of respiratory failure and acute renal failure increased between 2004–2005 and 2012–2013 from 18.8% to 32.6% (p = 0.0006) and from 9.9% to 28.1% (p < 0.0001), respectively. The frequency of chronic renal disease among ICU admissions with acute renal failure increased between 2004–2005 and 2012–2013 from 26.3% to 50% (p = 0.0600). Over the same period, use of mechanical ventilation and hemodialysis among all ICU admissions rose from 11.5% to 19.2% (p = 0.0216) and from 1.0% to 8.1% (p = 0.0007), respectively. The use of mechanical ventilation among those with respiratory failure and hemodialysis among those with acute renal failure did to not change significantly between 2004–2005 and 2012–2013, being 61.1% vs. 58.7% (p = 0.8004) for mechanical ventilation and 10.5% vs. 28.7% (p = 0.0992) for hemodialysis.

The median (interquartile range) hospital length of stay decreased between 2004–2005 and 2012–2013 from 12 (6–18) to 8 (4–16) days, respectively (-9.3%/2 years [-18.2% to -0.3%]), while the median adjusted total hospital charges remained unchanged (+5.0%/2 years [-9.6% to 19.5%]).

There was no significant change over time in short-term mortality (p = 0.9471), discharge home (p = 0.6942), or discharge to another facility (p = 0.1532).

### Predictors of short-term mortality

The findings on univariate analyses and those on covariates removed on backward analyses of multivariate logistic regression are provided in S3 Table and S4 Table, respectively. The results of multivariate analysis are detailed in Table 3. The model’s AUROC (95% CI) was 0.850 (0.814–0.886) and goodness-of-fit test showed X^2^ = 3.035 and p = 0.932. The predictive model showed that older ICU admissions and women had increased adjusted odds of short-term mortality, as were those receiving blood transfusion and developing increasing number of failing organ. Requirement of mechanical ventilation was the strongest predictor of short-term mortality among ICU admissions with aOR 7.982.

**Table 3:** Multivariate logistic regression of predictors of short-term mortality among ICU admissions.

Variables	Adjusted odds ratio (95% CI)	p value
**Age**		
< 45 years	1	
≥ 45 years	2.051 (1.231–3.415)	0.0058
**Gender**		
Male	1	
Female	1.907 (1.237–2.941)	0.0035
**Race/ethnicity**		0.0897
White	1	
Hispanic	1.142 (0.650–2.008)	
Black	0.344 (0.098–1.215)	
Other	1.942 (0.932–4.050)	
**Organ failures**		< 0.0001
0	1	
1	2.784 (1.605–4.831)	
2	3.662 (1.856–7.225)	
3+	5.427 (2.510–11.733)	
**Blood transfusion**	1.810 (1.125–2.911)	0.0144
**Mechanical ventilation**	7.982 (5.001–12.739)	< 0.0001
**Year of hospitalization**^**A**^	0.881 (0.766–1.014)	0.0582

A adjusted odds ratio is per 2-year period of hospitalization

None of the other examined socio-demographic factors, non-pulmonary comorbidities, or hospital-level characteristics predicted short-term mortality on adjusted analyses.

Although there was a significant interaction between age and gender on univariate analysis (p = 0.082), it was insignificant on adjusted analysis (p = 0.1140). On the exploratory subgroup analyses, short-term mortality was markedly higher among female ICU admissions younger than 45 years than that among their age group-similar male counterparts (14.1% vs. 7.4%, respectively; p = 0.0009). On the other hand, short-term mortality was similar between female and male ICU admissions aged 45 years and older (16.4% vs. 16.8%, respectively; p = 0.9270).

## Discussion

In this population-based cohort study we found increasingly high ICU utilization among adults with CF in Texas, with ICU admissions becoming sicker, showing rise in the burden of comorbidities and development of organ failure. The increased volume of ICU admissions was driven predominantly by older adults. Nevertheless, most ICU admissions and those requiring mechanical ventilation survived hospitalization with the majority of the reminder were discharged home. Adjusted odds of short-term mortality were higher among women, with older age, increasing number of organ failures and need for blood transfusion and mechanical ventilation.

We found high and rising burden of critical illness among adults with CF in Texas, averaging nearly 1 ICU admission per 5 adults per year in the state, by the end of study period. The burden of critical illness among adults with CF can be put in perspective by the finding that their incidence of ICU admission in the present study is over 7-fold higher than that in the general population in the US [33].

Our finding of over 3-fold rise in rates of ICU admissions with increasing age among CF hospitalizations likely reflects the noted corresponding incremental burden of comorbidities and number of organ failures. These findings indicate that with increasing aging of CF population, ICU utilization is expected to rise substantially.

Organ failures affected predominantly the respiratory and renal systems. However, unlike the expected predominance of respiratory failure among CF ICU admissions with organ failure, acute renal failure, shown to rise nearly 3-fold during study period, has not been generally associated with cystic fibrosis, including critical illness events. While the proximate causes of acute renal failure could not be ascertained from administrative data, the corresponding rapid rise in reported chronic renal disease was likely among the key contributors.

Prior studies of consecutive ICU admissions of adults with CF showed that 74% [6] to 90% [11, 16] of patients were admitted for respiratory crises. In contrast, a key finding of our study is that the majority of adults with CF admitted to ICU in Texas did not have acute respiratory system failure. Although specific indications for ICU admission were not available in the TIPUDF data, the later finding suggests marked population-level broadening of the spectrum of ICU admissions among adults with CF, and may explain in part the relatively low short-term mortality in our cohort. Further population-based studies are needed to examine the contemporary patient mix of critically ill adults with CF in other healthcare settings.

Only limited data were reported on measures of hospital resource utilization among ICU-managed adults with CF [13, 14, 16]. The marked decrease in hospital length of stay in our cohort, without concomitant decline in severity of illness or rise in discharge to other facilities, suggests increase in care efficiency.

The hospital-level fiscal footprint of hospitalizations with ICU admission of adults with CF has not been previously reported. The mean hospital charges per ICU admission for the whole cohort were nearly 4-fold higher than those per hospitalization in the general population in Texas in 2013 [34] and, when adjusted for inflation, over 2-fold higher than those per ICU admission in the general population in the US [35]. Importantly, the unchanged adjusted hospital charges over time, coupled with decreased hospital length of stay suggest marked increase in daily care intensity in our cohort.

The short-term mortality in our cohort is among the lowest reported among critically ill adults with CF, with prior studies describing mortality ranging from 0% [13] to 73% [12]. Our findings also highlight the previously noted low use of hospice and palliative care services in general among CF patients [36, 37], underscoring future opportunities for practice improvement. Short-term mortality rose with age, likely reflecting the corresponding incremental burden of chronic illness and organ failure. Direct comparisons of mortality in our cohort with prior studies are challenging due to the noted predominance of single-center data, with most described patients treated during the 1990s and early 2000s [6, 11, 12, 14–16], and the markedly higher ICU bed availability in the US, as compared to other high-income countries [38]. Nevertheless, our study extends findings from prior reports showing a pattern toward improving outcomes among critically ill adults with CF.

In contrast with the relatively low mortality for the whole cohort, our findings on the short-term mortality among the subset of ICU admissions undergoing mechanical ventilation are within the range reported by the majority of prior studies, with mortality rates in these reports ranging from 32.3% [15] to 58% [11]. Although the study by Sood and colleagues [6] prompted a pronouncement in 2001 that critical care in patients with CF “is no longer an oxymoron” [7], significant debate remains about the use of mechanical ventilation in critically ill patients with CF. Indeed, some have suggested that as most critically ill patients with CF have end-stage disease, intensive care should not be considered and that it is only rarely appropriate “to ventilate patients with CF who have respiratory failure” [8]. Nevertheless, available data suggest that considerable progress was made in the short-term outcomes of mechanically ventilated adult CF patients since the often-cited study by Davis et al, which found near uniform fatality [5]. Our study demonstrates that the population-level short-term mortality in a heterogeneous cohort of adults with CF who received mechanical ventilation is comparable to that of patients with the acute respiratory distress syndrome in the general population [39], for whom there is no debate on the usefulness of mechanical ventilation.

The hospital disposition of survivors of critical illness has not been described in prior studies of adults with CF. About 3 out of 4 of ICU admissions in our cohort were discharged home. As importantly, nearly 2 in 3 of the survivors in the subgroup that received mechanical ventilation (that is, after excluding those who died in the hospital or were discharged to hospice) were discharged home, representing a markedly higher rate than that (46.6%) reported among hospital survivors of the acute respiratory distress syndrome [40]. Thus, as critical illness in adults with CF is no longer mostly a fatal event, emphasis in future studies should shift to address longer-term consequences of survivorship [41], including, as is increasingly recognized in the general population, related physical, cognitive, and mental health dysfunction [42], as well as trajectories of subsequent increased use of health care resources [43, 44] and lower long-term survival [44].

Only patient-level covariates were associated with short-term mortality in our cohort, being restricted to advanced age, gender and, as expected, incremental organ failure and use of life-support interventions, with need for mechanical ventilation having the strongest adverse impact.

Gender-related disparities in long-term survival continue to affect adult women with CF [4], despite the noted progress in patient care. Our study shows that this survival gap among women extends to short-term mortality among critically ill patients. However, although the interaction of gender and age was not significant on multivariate analysis, we found on exploratory analyses that the worse short-term mortality among women in our cohort was driven exclusively by those younger than 45 years. These later findings are in line with those reported recently by Nick and colleagues, who have showed that among long-term survivors of CF, the survival gap was no longer observed among older women [32].

The odds of adjusted short-term mortality, though not statistically significant, tended to decrease over time. This trend was contrary to an expected rise, as ICU admissions were getting older and with increasing occurrence of organ failure and need for life-support interventions. However, apparent improvements in short-term mortality may represent an underestimated death rate when critically ill patients are increasingly discharged to other facilities [45]. Because there was no significant change in rates of discharge to another facility during study period, the combined findings of lack of rise of adjusted odds of death and unchanged unadjusted short-term mortality in our cohort may reflect improving care of critically ill adults with CF over time.

The rarity of ICU admissions of critically ill adults with CF among the general population of the critically ill limits development of institutional and clinicians’ expertise, and may potentially favor larger hospitals and ICUs and those facilities with broad disease-specific expertise. However, hospital-level characteristics did not affect risk of short-term mortality in our cohort, though they are well-known to impact outcomes in the general ICU population [46].

Only a small minority of ICU admissions were managed at the CF Foundation’s accredited care center facilities. This finding reflects the geographical reality where the latter facilities (n = 11) [26] represent a small fraction of the hundreds of hospitals in Texas [17], while the state population is dispersed over a land area larger than France [20]. Although CF patients in Texas can have periodic outpatient follow-up at the CF Foundation’s accredited care centers, the noted geographical constraints can preclude ready close-by access to inpatient care at these facilities at times of acute severe health crises. Since the TIPUDF data set does not include information on the outpatient care of hospitalizations, the association between the location or quality indicators of outpatient care and the risk of critical illness and related outcomes in our cohort cannot be inferred.

The long-term outcomes of patients followed at the CF Foundation accredited care centers were not been systematically compared with those not followed in these centers. Nevetheless, we hypothesized that short-term mortality of critically ill patients may be more favorable among those managed in facilities that were part of the CF Foundation care center network. A positive short-term prognostic impact for critically ill patients managed in these facilities may have supported consideration of regionalization of critical care of adults with CF, in line with similar proposals for the general population [47]. The sources of the apparently neutral outcome impact of care in hospitals that were part of the CF Foundation accredited care center network cannot be inferred from administrative data. However, the overall short-term mortality in the present study has been among the lowest reported to date in this population, while the short-term mortality among those requiring mechanical ventilation has been well comparable to that reported in many referral centers [11, 16, 48]. Thus, a key message of the present study is that at a population level the examined hospital-level factors did not adversely affect short-term outcomes of critically ill adults with CF.

Although our model demonstrated good predictive performance, we cannot exclude effects of residual confounding on our findings, due to the observational, retrospective study design. In addition, although we examined a large cohort of ICU admissions, we cannot exclude lack of significant prognostic impact of some of the examined covariates due to small subgroup numbers and possibly resultant type II errors.

The key strengths of our study include general and longitudinal examination of a large, unselected, population-based cohort of ICU admissions, transcending local variability in patient-mix, care, and outcome patterns. In addition, the study focused on a state with a diverse population, with interrogation of a high-quality data set.

Nevertheless, our findings should be considered in the context of several important limitations, related to the retrospective study design and use of administrative data. As a result, hospitalizations with CF may have been improperly identified or under-identified. However, CF hospitalizations were identified using an approach similar to prior epidemiological studies [49, 50]. As noted earlier, the timing and indications for ICU admission were not available, nor were data on the clinical variables that have affected clinicians’ triage to ICU or use of mechanical ventilation or hemodialysis. In addition, although our models demonstrated excellent discrimination and good calibration for prediction of short-term mortality, lack of data on some of the key characteristics known to affect long–term outcomes of CF patients, such as pulmonary function state, specific CF transmembrane conductance regulator gene mutations or chronic colonization patterns of the respiratory tract may have affected our findings. Finally, although we studied a large heterogeneous population, our findings may not reflect the populations of ICU-managed adults with CF in the remainder of the US or internationally.

## Conclusions

In this first population-based study in the US, we demonstrate incrementally high burden of critical illness among adults with CF. Although ICU admissions were increasingly older and sicker, most, including those requiring mechanical ventilation, survived hospitalization, with the majority of survivors discharged home, suggesting considerable short-term benefits of critical care in the present cohort. Increased risk of short-term mortality was associated with select demographic characteristics, acute organ failure and need of life-support interventions, while examined socio-economic features, non-pulmonary comorbidities and hospital-level attributes did not affect risk of death. While clinicians invariably individualize care decisions on use of life-support interventions, our results support consideration of mechanical ventilation in critically ill adults with CF. Although challenging due to the small number of affected patients in individual facilities, further studies are needed in other healthcare settings to enhance our understanding of the changing landscape of the population of critically ill adults with CF.

## Supporting information

S1 Table ICD-9-CM codes used to identify selected comorbidities and ICU procedures. (DOCX)

S2 Table The biennial number of hospitalizations and ICU admissions of adults with cystic fibrosis in Texas, 2004–2013. (DOCX)

S3 Table Univariate logistic regression of predictors of short-term mortality among ICU admissions. (DOCX)

S4 Table Variables eliminated on backward stepwise selection for the multivariate logistic regression of predictors of short-term mortality of ICU admissions. (DOCX)
