# Supplementary material for: Critical illness among adults with cystic fibrosis in Texas, 2004–2013: Patterns of ICU utilization, characteristics, and outcomes
Source: PLoS One. 2017 Oct 24;12(10):e0186770. doi: 10.1371/journal.pone.0186770 (PMC5655478; doi:10.1371/journal.pone.0186770)
Supplement: S1 Table — (DOCX) [file pone.0186770.s001.docx]

**Critical Illness among Adults with Cystic Fibrosis in Texas, 2004-2013: Patterns of ICU utilization, Characteristics, and Outcomes**

**Lavi Oud, MD**

**S1 Table. International Classification of Diseases*,* Ninth Revision*,* Clinical Modification (ICD-9-CM) codes used to identify selected comorbidities and ICU procedures.** (Where only 3 or 4-digit codes are listed, all associated subcodes are included)

**Variable ICD-9-CM codes**

Depression 293.83, 296.2, 296.3, 296.4, 296.5, 296.6, 296.7, 296.8, 296.9, 300.4, 311

Anxiety 293.84, 300.0X, 300.2X, 300.3, 300.5, 300.89, 300.9, 308.1, 308.2, 308.4, 308.9, 309.81, 313.0, 313.1, 313.21, 313.22, 313.3, 313.82, 313.83

Alcohol abuse 291.XX, 303.XX, 305.0X, 357.5, 425.5, 535.3, 535.30, 535.31, 571.0, 575.1, 575.2, 575.3, 760.71, 980.0

Drug abuse 292.XX, 304.XX, 305.2X, 305.3X, 305.4X, 305.5X, 305.6X, 305.7X, 305.8X, 305.9X, 648.30, 648.31, 648.32, 648.33, 648.34, 655.50, 655.51, 655.53, 760.72, 670.73, 760.75, 779.5, 965.00, 965.01, 965.01, 965.09, V6542

Tobacco use 305.1X, V15.82

Mechanical ventilation 96.70-96.72

Hemodialysis 38.95, 39.95

Blood transfusion 99.00-99.09, V58.2
