# Supplementary material for: Critical illness among adults with cystic fibrosis in Texas, 2004–2013: Patterns of ICU utilization, characteristics, and outcomes
Source: PLoS One. 2017 Oct 24;12(10):e0186770. doi: 10.1371/journal.pone.0186770 (PMC5655478; doi:10.1371/journal.pone.0186770)
Supplement: S2 Table — (DOCX) [file pone.0186770.s002.docx]

**Critical Illness among Adults with Cystic Fibrosis in Texas, 2004-2013: Patterns of ICU utilization, Characteristics, and Outcomes**

**Lavi Oud, MD**

| **S2 Table. The biennial number of hospitalizations and ICU admissions of adults**  **with cystic fibrosis in Texas, 2004-2013** | | | | | | | | | | | |
| --- | --- | --- | --- | --- | --- | --- | --- | --- | --- | --- | --- |
|  |  |  |  |  |  |  |  |  |  |  |  |
| **Years** | | **Hospitalizations** | | **ICU admissions** | |  |  |  |  |  |  |
| 2004-2005 | | 1,581 | | 192 | |  |  |  |  |  |  |
| 2006-2007 | | 1,659 | | 216 | |  |  |  |  |  |  |
| 2008-2009 | | 1,921 | | 230 | |  |  |  |  |  |  |
| 2010-2011 | | 2,108 | | 277 | |  |  |  |  |  |  |
| 2012-2013 | | 2,310 | | 334 | |  |  |  |  |  |  |
