# Supplementary material for: Critical illness among adults with cystic fibrosis in Texas, 2004–2013: Patterns of ICU utilization, characteristics, and outcomes
Source: PLoS One. 2017 Oct 24;12(10):e0186770. doi: 10.1371/journal.pone.0186770 (PMC5655478; doi:10.1371/journal.pone.0186770)
Supplement: S3 Table — (DOCX) [file pone.0186770.s003.docx]

**S3 Table . Univariate logistic regression of predictors of short-term mortality among ICU admissions**

| **Variables** | | | **Adjusted odds ratio (95% CI)** | | | **p value** |
| --- | --- | --- | --- | --- | --- | --- |
| **Age** |  |  |  |  |  | 0.0232 |
| < 45 years | |  | 1 | | |  |
| ≥ 45 years | |  | 2.051 (1.231-3.415 | | |  |
| **Gender** |  |  |  |  |  | 0.0044 |
| Male |  |  | 1 | | |  |
| Female |  |  | 1.907 (1.237-2.941) | | |  |
| **Race/ethnicity** | |  |  |  |  |  |
| White |  |  | 1 | | |  |
| Hispanic |  |  | 1.142 (0.650-2.008) | | | 0.4099 |
| Black |  |  | 0.344 (0.098-1.215) | | | 0.0768 |
| Other |  |  | 1.942 (0.932-4.050) | | | 0.0694 |
| **Health insurance** | |  |  |  |  |  |
| Private |  |  | 1 | | |  |
| Medicare |  |  | 1.017 (0.660-1.567) | | | 0.9357 |
| Medicaid |  |  | 0.824 (0.524-1.296) | | | 0.4030 |
| Uninsured |  |  | 0.778 (0.321-1.881) | | | 0.5778 |
| Other |  |  | 0.616 (0.142-2.671) | | | 0.5176 |
| **Median income** | | |  | | |  |
| Quartile 1 (lowest) | |  | 1 | | |  |
| Quartile 2 | | | 1.170 (0.799-1.713) | | | 0.4173 |
| Quartile 3 | | | 1.379 (0.673-2.824) | | | 0.3793 |
| Quartile 4 (highest) | | | 0.727 (0.092-5.721) | | | 0.7622 |
| **Rural residence** | |  |  |  |  |  |
| No |  |  | 1 | | |  |
| Yes |  |  | 1.192 (0.710-1.999) | | | 0.5058 |
| **Comorbidities** | |  |  |  |  |  |
| Myocardial infarction | | | 0.366 (0.049-2.747) | | | 0.3289 |
| Congestive heart failure | | | 2.418 (1.453-4.025) | | | 0.0007 |
| Peripheral vascular disease | | | 1.064 (0.314-3.602) | | | 0.9201 |
| Cerebrovascular disease | | | 1.659 (0.556-4.950) | | | 0.3634 |
| Rheumatic disease | |  | 0.864 (0.108-3.764) | | | 0.8460 |
| Liver disease | |  | 0.946 (0.545-1.641) | | | 0.8438 |
| Diabetes |  |  | 0.750 (0.485-1.159) | | | 0.1960 |
| Hemiplegia | |  | 1.816 (0.511-6.452) | | | 0.3562 |
| Renal disease | |  | 0.738 (0.376-1.449) | | | 0.3780 |
| Malignancy | |  | 2.340 (0.849-6.443) | | | 0.0999 |
| Metastatic disease | |  | 5.257 (0.870-31.735) | | | 0.0704 |

**S3 Table . Univariate logistic regression of predictors of short-term mortality among ICU admissions (cont.)**

| Depression | |  | 0.884 (0.547-1.430) | | | 0.6178 |
| --- | --- | --- | --- | --- | --- | --- |
| Anxiety |  |  | 1.089 (0.604-1.962) | | | 0.7754 |
| Alcohol abuse | |  | 0.437 (0.104-1.839) | | | 0.2592 |
| Drug abuse | |  | 0.422 (0.152-1.176) | | | 0.0991 |
| Tobacco use | |  | 0.877 (0.445-1.729) | | | 0.7053 |
| Malnutrition | |  | 1.439 (0.999-2.075) | | | 0.0505 |
| **Type of hospitalization** | | |  |  |  |  |
| Surgical |  |  | 1 | | |  |
| Medical |  |  | 0.741 (0.504-1.091) | | | 0.1297 |
| **Organ failures** | |  |  |  |  |  |
| 0 |  |  | 1 | | |  |
| 1 |  |  | 4.991 (3.064-8.131) | | | < 0.0001 |
| 2 |  |  | 8.993 (5.060-15.982) | | | < 0.0001 |
| 3+ |  |  | 18.871 (10.141-35.113) | | | < 0.0001 |
| **Mechanical ventilation** | | | 13.312 (9.031-19.622) | | | < 0.0001 |
| **Hemodialysis** | |  | 2.223 (1.173-4.214) | | | 0.0143 |
| **Blood transfusion** | |  | 3.449 (2.354-5.039) | | | < 0.0001 |
| **Hospital characteristics** | | |  |  |  |  |
| **Bed number** | |  |  |  |  |  |
| < 200 |  |  | 1 | | |  |
| 200-399 |  |  | 1.034 (0.502-2.129) | | | 0.9266 |
| 400-599 |  |  | 1.288 (0.666-2.491) | | | 0.4517 |
| ≥ 600 |  |  | 1.076 (0.551-2.098) | | | 0.8295 |
| **ICU capacity (%)** | |  |  |  |  |  |
| < 10 |  |  | 1 | | |  |
| 10-15 |  |  | 1.122 (0.720-1.749) | | | 0.6100 |
| ≥ 15 |  |  | 0.638 (0.351-1.160) | | | 0.1412 |
| **Teaching status** | |  |  |  |  |  |
| No |  |  | 1 | | |  |
| Yes |  |  | 1.389 (0.975-1.979) | | | 0.0688 |
| **CFF Care Center^a^** | |  |  |  |  |  |
| No |  |  | 1 | | |  |
| Yes |  |  | 0.765 (0.436-1.343) | | | 0.3513 |
| **Rural hospital** | |  |  |  |  |  |
| No |  |  | 1 | | |  |
| Yes |  |  | 1.305 (0.379-4.489) | | | 0.672 |
| a Facilities that are part of Cystic Fibrosis Foundation Care Center Network | | | | | | |
|  | | | | | | |
