# Supplementary material for: Critical illness among adults with cystic fibrosis in Texas, 2004–2013: Patterns of ICU utilization, characteristics, and outcomes
Source: PLoS One. 2017 Oct 24;12(10):e0186770. doi: 10.1371/journal.pone.0186770 (PMC5655478; doi:10.1371/journal.pone.0186770)
Supplement: S4 Table — (DOCX) [file pone.0186770.s004.docx]

**Critical Illness among Adults with Cystic Fibrosis in Texas, 2004-2013: Patterns of ICU utilization, Characteristics, and Outcomes**

**Lavi Oud, MD**

**S4 Table. Variables eliminated on backward stepwise selection for the multivariate logistic regression of predictors of short-term mortality of ICU admissions**

| **Variables** |  |  | **p value** |
| --- | --- | --- | --- |
| Congestive heart failure | | | 0.3429 |
| Malignancy | |  | 0.1706 |
| Metastatic disease | |  | 0.9881 |
| Drug abuse | |  | 0.9854 |
| Malnutrition | |  | 0.9498 |
| Hemodialysis | |  | 0.7513 |
| Teaching status | |  | 0.304 |
